# Effects of Multi-Component Exercise on Sleep Quality in Middle-Aged Adults

**DOI:** 10.3390/ijerph192315472

**Published:** 2022-11-22

**Authors:** Jing-Yi Ai, Garry Kuan, Linda Ya-Ting Juang, Ching-Hsiu Lee, Yee-Cheng Kueh, I-Hua Chu, Xiao-Ling Geng, Yu-Kai Chang

**Affiliations:** 1 Department of Physical Education and Sport Sciences, National Taiwan Normal University, Taipei 106209, Taiwan; 2 Exercise and Sports Science Programme, School of Health Sciences, Universiti Sains Malaysia, Kubang Kerian 16150, Malaysia; 3 Lifestyles of Health and Sustainability Executive Master of Business Administration, National Taiwan Normal University, Taipei 106209, Taiwan; 4 Biostatistics and Research Methodology Unit, School of Medical Sciences, Universiti Sains Malaysia, Kubang Kerian 16150, Malaysia; 5 Department of Sports Medicine, College of Medicine, Kaohsiung Medical University, Kaohsiung 807, Taiwan; 6 Department of Medical Research, Kaohsiung Medical University Hospital, Kaohsiung 807, Taiwan; 7 College of Medicine, Kaohsiung Medical University, Kaohsiung 807, Taiwan; 8 Department of Wushu, Hong Kong Sports Institute, Hong Kong 999077, China; 9 Institute for Research Excellence in Learning Science, National Taiwan Normal University, Taipei 106209, Taiwan

**Keywords:** multi-component exercise, sleep quality, physical fitness, middle-aged adults

## Abstract

Sleep is a crucial factor in healthy aging. However, most middle-aged adults experience high levels of sleep disorders. While previous findings have suggested exercise training could benefit the quality of sleep, the effects of multi-component exercise on sleep quality are less examined. Accordingly, the current study aimed to assess the effectiveness of a multi-component exercise program on the quality of sleep among middle-aged adults. Twenty-four middle-aged adults were randomly assigned either to a multi-component exercise (MCE) group or a control group. The participants in the MCE group attended a 90-min session per week for 12 weeks. The control group was instructed to maintain their daily routine for 12 weeks. The primary outcome was the sleep quality evaluated by the Pittsburgh Sleep Quality Index (PSQI). The secondary outcome was physical fitness, including muscular strength and endurance, balance, and flexibility. Regarding sleep quality, the global mean score (*p* = 028), sleep disturbances (*p* = 011), and sleep efficiency (*p* = 035) of the PSQI scores were significantly reduced in the MCE group after the 12-week intervention. Regarding physical fitness, the flexibility of the MCE group improved significantly after the intervention (*p* = 028), yet, no significant change was observed in the control group. Additionally, the muscular strength of the control group declined significantly after the 12-week period (*p* = 034). Our results revealed the effectiveness of the MCE intervention in improving sleep quality and physical fitness in middle-aged adults. Further studies using larger sample sizes, objective measures of sleep quality, different types of exercise training, as well as different populations, are warranted to extend our current findings.

## 1. Introduction

Sleep is a crucial factor in healthy aging [1]. Good sleep could enhance cognition, mental health, ability to engage in activities, self-reported health, and reduce fragility [2]. Aging is associated with declines in most physiological systems that culminate in sleep changes and limited physical function [2,3,4]. A higher prevalence of poor sleep quality is reported among the adult population, and aging is associated with drowsiness and physical disabilities [5,6]. Therefore, it is critical to establish strategies for improving sleep quality and physical function in adults. Medication or passive treatment, such as benzodiazepines, sleep restriction, and sleep hygiene treatments, have been commonly used by adults for sleep disorders, but long-term usage of these medications poses serious adverse health effects [7]. 

Exercise has been suggested as a low-risk [8], non-pharmacological intervention to improve aspects of sleep (e.g., sleep quality, sleep disturbance, and sleep efficiency) [9,10,11]. Recent research shows that participating in exercise programs positively affects sleep quality [8]. The potential mechanisms by which exercise improves sleep may be melatonin-mediated factors, improved mood [12], or an increased brain-derived neurotrophic factor level [13]. However, while most studies examining the effects of exercise on sleep have focused on either sleep disorders or special populations (e.g., postmenopausal women and individuals with obstructive sleep apnea), few studies have targeted individuals with subclinical sleep problems. This population may also improve their sleep quality by engaging in exercise. 

The Physical Activity Guideline Advisory Committee and American College of Sports Medicine [14,15] have recommended multi-component exercise combining endurance, strength, balance, flexibility training, and meditative attention [16] to promote physical health and avoid injuries caused by the inequality between the improvement of a single component and ignoring the other [14]. Positive associations between multi-component exercise and sleep quality have recently been reported [17]. Multi-component exercises may directly or indirectly affect sleep and physical health through multiple factors, such as physical fitness (endurance, strength, balance, flexibility) [16], as well as mental and social factors [18]. A recent meta-analysis reported that multi-component exercises might have specific effects on the quality of sleep [17,19]. Furthermore, multi-component exercises involving mind-body practice, such as Tai Qi, Pilates, Yoga, and Feldenkrais [20,21,22,23] may enhance sleep quality beyond that of traditional physical exercise [24,25,26]. The multi-component exercise that involves social interaction can also improve mood and benefit sleep by reducing feelings of loneliness [27]. However, studies that examined the effects of such exercise programs on middle-aged adults are few, and the results are inconclusive. Considering the high prevalence of sub-clinical sleep disturbances in middle-aged adults, it is important to assess the long-term effects of regular multi-component exercise on sleep quality in this population.

The current study aimed to investigate the effects of multi-component exercise on sleep quality and physical fitness among middle-aged adults. We hypothesized that participants who underwent a 12-week multi-component exercise program would show improvements in sleep quality and physical fitness.

## 2. Materials and Methods

### 2.1. Participant

We recruited participants from the communities in Taipei, Taiwan. Healthy participants who met the following inclusion criteria were recruited: (1) 40–60 years of age; (2) no contraindications to exercise; (3) have not been involved in any experimental research in the last year. The participants who had the following conditions were excluded from this study: (1) physical disability that prevents participants from exercising; (2) regular practice of exercise or mindfulness; and (3) major confounding factors known to cause clinical sleep disturbances (e.g., mild cognitive impairment, autoimmune disease, obstructive sleep apnea syndrome).

### 2.2. Study Design

This study used a quasi-experimental design. The participants were randomly assigned to either the multi-component exercise or the control group. Outcome measures were conducted at baseline and after the 12-week intervention period. This study was reviewed and approved by the Institutional Review Board of the National Taiwan Normal University. A written informed consent form was given to and signed by all participants.

### 2.3. Intervention

#### 2.3.1. Multi-Component Exercise Group (MCE)

The intervention was a theory-based 12-week supervised multi-component exercise program entitled Bagua Daoyins. This unique program was designed based on brain science and an Asian traditional martial arts culture and was taught through a progressive training approach. Participants in the MCE group attended a 90-min exercise session per week for 12 weeks. Each session consisted of a warm-up and recovery routine, physical fitness exercises (i.e., muscular strength and endurance, balance, and flexibility), meditation, and social interactions. An experienced and certified fitness instructor supervised the training program. Participants were trained in a group of 12 persons.

#### 2.3.2. Control Group

Participants in the control group were instructed to maintain their daily routines. No exercise program or any behavioral management training was assigned to this group.

### 2.4. Outcome Measures

#### 2.4.1. Pittsburgh Sleep Quality Index (PSQI)

The Pittsburgh Sleep Quality Index (PSQI) is a widely utilized self-assessed questionnaire for sleep quality [28]. The questionnaire consists of 19 questions assessing sleep quality over the past 30-day interval [28]. The PSQI has seven sub-components (i.e., sleep quality, sleep latency, sleep duration, habitual sleep efficiency, sleep disorders, sleep medication use, and daytime dysfunction due to sleepiness), each with a scale ranging from 0 to 3. The total PSQI score ranges from 0 to 21, with higher scores indicating poorer sleep quality. Scores ≧ 5 were considered indicative of poor sleep quality. The PSQI has good internal consistency and test-retest reliability [29].

#### 2.4.2. Physical Fitness

Four elements of physical fitness (i.e., muscular strength and endurance, flexibility, balance, and body mass index) were examined.

Muscular strength was assessed using a hand grip dynamometer (Takei, Niigata, Japan). The measurement was performed in a sitting position, with the subject seated on a chair without armrests, and with the feet of the examined person resting flat on the floor, arms set along the torso, the elbow flexed at 90°, the forearm in a neutral position, and the wrist in a position from 0° to 30° of extension. The participant was instructed to clench the hand maximally and hold the grip tightly for 6 s. The procedure was repeated three times for the dominant hand, with a one-minute rest between the tests. The average of three measurements (in kilograms) was recorded [30].

Muscular endurance was assessed via the sit-up test [31]. Bent knee sit-ups were used to test the local endurance of the participants’ abdominal muscles. The procedure that was followed required that the subject interlace the fingers over the back of the head while lying supine with the knees bent at 90° or less, and with the ankles supported by assistants. The movement was accomplished by the subject sitting up and simultaneously touching both elbows to the knees and then returning to the starting position. The score was the number of legal sit-ups that were completed in one minute. Two trials were allowed, and the better of the two scores was recorded.

Flexibility was assessed via the sit-and-reach test by using a sit-and-reach box [32]. Participants sat barefoot with their feet placed flat against the box. With their hands together, the participants slowly reached forward as far as possible while keeping their knees extended. Two trials were performed, and the best score was recorded in cm to the first decimal place.

Balance was assessed using the single-leg stance test with the eyes closed [33]. Participants were asked to stand barefoot on the limb of their choice, with the other limb raised so that the raised foot was near but not touching the ankle of their stance limb. The participants were instructed to cross their arms over their chests and maintain their balance on one leg while keeping their eyes closed. The investigator started the stopwatch as soon as the participants lifted their chosen foot off the floor. The test was terminated when the participants either: (1) uncrossed their arms, (2) moved the raised foot toward or away from the standing limb or touched the floor, (3) moved the weight-bearing foot to maintain balance, (4) exceeded the maximum duration of 45 s, or (5) opened his/her eyes. The participants performed this test third in the sequence, and the average time for the 3 trials was recorded.

Body mass index (BMI) was calculated from the participant’s height and weight. Each participant’s height was assessed by using a stadiometer, and their weights were individually assessed by using a bioelectrical impedance analysis device (BC-418, Tanita Corp., Tokyo, Japan).

#### 2.4.3. Data Analysis

Descriptive statistics (mean and standard deviation) were calculated to summarize the characteristics of the participants. Sleep quality-related indices (i.e., PSQI global score, subjective sleep quality, daytime dysfunction, use of sleeping medications, sleep disturbances, sleep latency, sleep efficiency, and total sleep time) and physical fitness indices (i.e., muscular strength and endurance, flexibility, balance, and BMI) were separately analyzed using a 2 (MCE vs. control) × 2 (pre-test vs. post-test) mixed design analysis of variance (ANOVA). Post-hoc comparisons were conducted with Bonferroni correction. An estimate of effect size, using the partial eta squared (η^2^), was reported to present the magnitude of the effect. Significance was set at *p* < 05. Statistical analyses were conducted using the SPSS 20.0 version (SPSS Inc., Chicago, IL, USA).

## 3. Results

### 3.1. Characteristics

Twenty-four participants were recruited and assigned to either the MCE group (*n* = 12) or the control group (*n* = 12). Baseline descriptive statistics are presented in Table 1.

None of the baseline variables were significantly different between the two groups. The baseline PSQI scores of the MCE group and the control group were 9.0 ± 4.2 and 6.7± 2.9, respectively, indicating moderate and greater sleep problems.

### 3.2. Pittsburgh Sleep Quality Index (PSQI)

**Figure 1:**
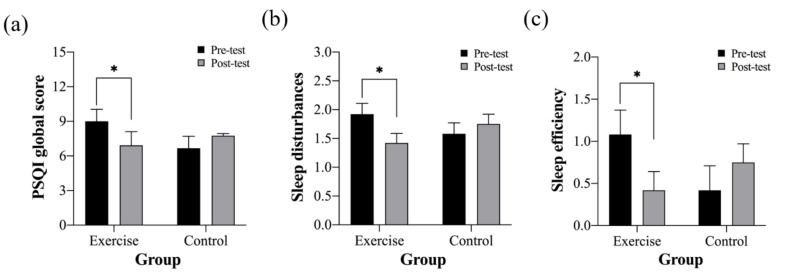
Changes in the Pittsburgh Sleep Quality Index (PSQI) scores over time between multi-component exercise (exercise) and control groups: (**a**) PSQI global score; (**b**) sleep disturbances; (**c**) sleep efficiency. * Significant difference between the pre-test and the post-test. Note. Data are presented as standard errors of mean.

The baseline average PSQI global score was 7.83 ± 3.70. The PSQI scores of the two groups are presented in Figure 1 and Table 2. The interaction effects were significant in the PSQI global score (F (1, 22) = 6.37, *p* = 019, η2 = 23), the sleep disturbance score (F (1, 22) = 6.77, *p* = 016, η2 = 24), and the sleep efficiency score (F (1, 22) = 5.66, *p* = 026, η2 = 21). The PSQI global score of the MCE group was reduced significantly from the pre-test to the post-test (*p* = 028). Similarly, sleep disturbance (*p* = 011) and sleep efficiency (*p* = 035) of the MCE group were reduced significantly from the pre-test to the post-test. No other significant change in the PSQI parameters was observed in the MCE group. No significant change in the PSQI parameters was observed in the control group.

### 3.3. Physical Fitness

**Figure 2:**
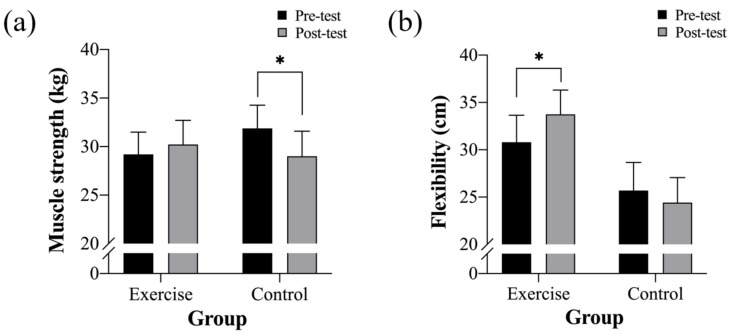
Changes in physical fitness over time between multi-component exercise (exercise) and control groups: (**a**) muscle strength and (**b**) flexibility. * Significant difference between the pre-test and the post-test.

The physical fitness of the two groups is presented in Figure 2 and Table 3. A significant interaction effect was observed in the flexibility (F(1, 22) = 5.36, *p* = 029, η^2^ = 18). The flexibility improved significantly at the post-test in the MCE group (*p* = 028). Furthermore, an interaction effect was observed in the grip strength (F(1, 22) = 4.74, *p* = 039, η^2^ = 16). The grip strength reduced significantly from the pre-test to the post-test in the control group (*p* = 034). No significant changes were observed in other physical fitness parameters in either group.

## 4. Discussion

The results of the current study showed that a 12-week MCE program was effective in improving sleep quality (global sleep quality, sleep disturbances, and sleep efficiency) and flexibility and preventing a decline of muscular strength in middle-aged adults. 

### 4.1. Effects of the Intervention on Sleep Quality

Our training program adopted a novel form of multi-component exercise, which included physical fitness, slow and gentle diaphragmatic breathing, and relaxation, and was shown to improve several sleep components in middle-aged adults. These findings are consistent with a meta-analysis reporting that different types of exercise, including single-component exercise and combined exercise, were associated with improvements in adults’ PSQI [34].

The frequency, duration, and intensity of exercise are also key factors proposed to affect the quality of sleep. Vanderlinden et al. [8] reported that an exercise frequency of three times per week showed significantly more benefits in sleep outcome measures, when compared to a higher frequency (once per day) and a lower frequency (once per week). On the other hand, the American National Sleep Foundation recommends applying regular aerobic exercise (>150 min/week to improve sleep quality) [35]. Our MCE program was performed at a lower frequency (once per week). While this training frequency was effective in improving sleep quality in the present study, a higher frequency (e.g., 2–3 times per week) may render more benefits and thus warrants further investigation.

In terms of the duration of exercise training, our results conform to findings from a recent meta-analysis indicating that a 12-week exercise training could have significantly more positive impacts on the quality of sleep [34]. This meta-analysis also showed that an exercise program of more than 12 weeks had a greater effect on the improvement of sleep quality than an exercise program of few than 12 weeks. Additionally, it was shown that intensity lower-intensity of exercise might be more suitable for middle-aged adults, due to lower risk of injuries, better compliance, and longer sustainability [36]. However, in the present study, the indicators of intensity were not assessed and should be included in future studies.

While exercise has been shown to be beneficial for sleep, the underlying mechanisms remain unclear. One of the proposed mechanisms is that physical activity can change melatonin levels [37]. Melatonin is a key hormone that regulates the circadian rhythm and sleep [38]. It was shown that exercise can modulate melatonin levels [20,39]. Previous studies also suggested that yoga in the MCE can be used as psychophysiological stimuli for increasing the secretion of melatonin, which could be responsible for improving the quality of sleep [20]. In the current study, our MCE program may have increased the melatonin levels in participants through slow and gentle diaphragmatic breathing and relaxation (common techniques practiced in a yoga session), resulting in better sleep quality.

Chronic exercise may also influence the endocrine and metabolic systems. Our 12-week MCE program may have improved sleep quality via changes in glucose metabolism and the release of BDNF, which are potential mechanisms leading to sleep impairment [40]. Besides, the MCE might improve sleep quality by modulating the autonomic nervous system [41]. Our MCE program included extensive postures and controlled breathing. These techniques are reported to reduce sympathetic nerve activity, enhance parasympathetic nerve activity, and improve physiological reactivity to stress [42]. Such changes could also contribute to improvements in sleep [43]. These results suggest that multi-component exercise may also play an important role in modulating the neuroendocrine and autonomic nervous systems, factors that may improve sleep habits. Future studies that examine these underlying mechanisms in middle-aged adults are warranted.

Our MCE program also included meditation and social interaction, which may offer additional benefits. Meditation involves awareness and acceptance, which may help disengage from ruminative thoughts and daily stress, thereby reducing mental stress and arousal and improving sleep quality [44]. Mind-body exercise that combines body movement and meditation (e.g., yoga, tai chi) was shown to improve subjective sleep quality [17,19]. The MCE that involves meditative movement, which means paying attention in a particular way [23], was also found to mitigate emotional reactivity [45] and promote impartial reappraisal of the salient experiences, which together may facilitate sleep [26]. Therefore, an MCE program that combines meditation might have a special mechanistic route to improved sleep, beyond that of a component single-component exercise [24,25]. Social interaction could also benefit sleep. A previous study showed that the elderly with good social involvement have a better quality of sleep [46]. This advantage has been attributed to a greater feeling of belonging and social inclusion, achieved by sharing time and engaging in group activities [46]. In the present study, the MCE was conducted in a group of 12 persons that provided opportunities for social interaction. This may promote social bonds and reduce feelings of loneliness, which could result in the release of the neuropeptide oxytocin that further improves sleep quality [47,48].

### 4.2. Effects of the Intervention on Physical Fitness

The present study demonstrated that the MCE not only positively affected sleep quality but also improved physical fitness. It was found to improve flexibility and prevent the decline of muscular strength as a result of aging.

Flexibility, as a key element of fitness, refers to the internal characteristics of the body tissue, which ensure the maximum range of joint movement without injury [49]. The beneficial effects on flexibility may be due to the repetitive stretching in the MCE. Our MCE program included practices of holding the stretching poses for a period of time with controlled breathing. These techniques have been shown to be effective in increasing flexibility [50] and thus may have contributed to the improvements in flexibility found in the present study.

Maintaining muscle mass and function in middle age is important [51]. In the present study, muscle strength was found to decrease in the control group, while no change was observed in the MCE group, suggesting that the MCE program may help maintain muscle strength and prevent the decline in physical fitness associated with aging [52]. Our MCE program included practices of holding static postures and moving in a controlled pattern from one posture to another. These practices may have facilitated the maintenance of muscular strength. Our findings are consonant with the results of 12-week multi-model exercises that combined dance and yoga. This program was shown to be effective in improving muscle strength, flexibility, functional balance, and mobility [53]. It is thus suggested that an MCE program may be considered an appropriate intervention for enhancing physical fitness, which could contribute to delaying and preventing frailty in middle-aged adults [54].

### 4.3. Limitations and Directions for Future Research

There are several limitations to be noted. First, this trial had a short intervention period without follow-up. Therefore, the long-term effects of the MCE program are unknown. Second, due to the characteristics of an MCE program, the isolated impact of each component of the MCE program could not be assessed (i.e., physical fitness, meditation, or social interaction). Third, our sample size was relatively small, which did not provide enough strength of value (0.46) for detecting a difference among the groups. In addition, as the male sample was very limited in the current study, so the results may not be generalized to this gender. Future studies with larger sample sizes and more male participants are warranted. Fourth, the inclusion of another exercise group in the study design is needed in order to further compare the effects of MCE to other types of exercises that have been previously shown to benefit sleep quality. Fifth, measures of the biomarkers of neurological function (e.g., BDNF), heart rate variability, and psychological status are warranted to further examine the potential underlying mechanisms responsible for the beneficial effects of the MCE on sleep quality.

## 5. Conclusions

The current study showed that 12 weeks of novel multi-component exercise training was effective in enhancing sleep quality and physical fitness in middle-aged adults. Our findings provide support for the multi-component exercise training as a new strategy for health promotion in this population.

## Figures and Tables

**Table 1:** Sample characteristics (M ± SD).

	All Participants (24)	Multi-Component Exercise (*n* = 12)	Control (*n* = 12)
Gender (male/female)	1.83 ± 0.38	2/10	2/10
Age (years)	54.7 ± 7.54	54.17 ± 6.70	55.25 ± 8.57
Height (cm)	160.71 ± 5.44	160.96 ± 5.28	160.46 ± 5.81
BMI (kg/m^2^) PSQI global score	26.40 ± 4.18 7.83 ± 3.70	24.22 ± 3.50 9.00 ± 4.16	26.52 ± 3.00 6.67 ± 2.90

Note. Data are presented as mean ± standard deviation. BMI = body mass index.

**Table 2:** Changes in the Pittsburgh Sleep Quality Index (PSQI) scores over time.

	Multi-Component Exercise (*n* = 12)		Control (*n* = 12)	
PSQI	Pre-Test	Post-Test	Pre-Test	Post-Test
PSQI global score	9.00 ± 4.16	6.92 ± 4.10 *	6.67 ± 2.90	7.75 ± 4.14
Subjective sleep quality	1.58 ± 0.79	1.08 ± 0.79	1.42 ± 0.79	1.42 ± 0.79
Daytime dysfunction	1.25 ± 0.75	0.75 ± 0.62	1.00 ± 0.74	0.92 ± 0.90
Use sleeping medications	0.58 ± 1.17	0.67 ± 1.12	0.00 ± 0.00	0.42 ± 1.00
Sleep disturbances	1.92 ± 0.79	1.42 ± 0.52 *	1.58 ± 0.52	1.75 ± 0.62
Sleep latency	1.50 ± 1.09	1.25 ± 1.06	1.50 ± 0.80	1.25 ± 0.87
Sleep efficiency	1.08 ± 1.24	0.42 ± 0.67 *	0.42 ± 0.67	0.75 ± 0.87
Total sleep time	1.08 ± 0.90	0.83 ± 0.72	1.08 ± 0.67	1.42 ± 0.79

Note. Data are presented as mean ± standard deviation. * *p* < 05.

**Table 3:** Changes in physical fitness over time.

	Multi-Component Exercise (*n* = 12)		Control (*n* = 12)	
Physical Fitness	Pre-Test	Post-Test	Pre-Test	Post-Test
Muscle strength (kg)	29.21 ± 9.14	30.22 ± 10.20	31.88 ± 7.92	29.00 ± 8.25 *
Muscle endurance (reps)	16.00 ± 10.26	16.27 ± 10.07	12.05 ± 6.46	13.63 ± 7.15
Balance (s)	14.36 ± 13.90	24.79 ± 25.11	10.85 ± 9.14	16.43 ± 20.89
Flexibility (cm)	30.79 ± 11.06	33.75 ± 8.51 *	25.69 ± 10.38	24.42 ± 10.60

Note. Data are presented as mean ± standard deviation. * *p* < 05.

## Data Availability

The data is available upon request from the authors.
